# IolR, a negative regulator of the *myo*-inositol metabolic pathway, inhibits cell autoaggregation and biofilm formation by downregulating RpmA in *Aeromonas hydrophila*

**DOI:** 10.1038/s41522-020-0132-3

**Published:** 2020-05-20

**Authors:** Yuhao Dong, Shougang Li, Dan Zhao, Jin Liu, Shuiyan Ma, Jinzhu Geng, Chengping Lu, Yongjie Liu

**Affiliations:** Joint International Research Laboratory of Animal Health and Food Safety, College of Veterinary Medicine, Nanjing Agricultural University, Nanjing, 210095 China

**Keywords:** Biofilms, Pathogens, Biofilms, Pathogens

## Abstract

*Aeromonas hydrophila* is the causative agent of motile Aeromonad septicemia in fish. Previous studies have shown that the *myo*-inositol metabolism is essential for the virulence of this bacterium. IolR is a transcription inhibitor that negatively regulates *myo*-inositol metabolic activity. While in the process of studying the inositol catabolism in *A. hydrophila* Chinese epidemic strain NJ-35, we incidentally found that *ΔiolR* mutant exhibited obvious autoaggregation and increased biofilm formation compared to the wild type. The role of surface proteins in *A. hydrophila* autoaggregation was confirmed by different degradation treatments. Furthermore, calcium promotes the formation of aggregates, which disappear in the presence of the calcium chelator EGTA. Transcriptome analysis, followed by targeted gene deletion, demonstrated that biofilm formation and autoaggregation caused by the inactivation of *iolR* was due to the increased transcription of a RTX-family adhesion gene, *rmpA*. Further, IolR was determined to directly regulate the transcription of *rmpA*. These results indicated that *iolR* is negatively involved in autoaggregation and biofilm formation in *A. hydrophila*, and this involvement was associated with its inhibition on the expression of *rmpA*.

## Introduction

*Aeromonas hydrophila* is a gram-negative ubiquitous bacterium that causes infections in humans and a wide variety of aquatic and terrestrial animals^1^. The pathogenesis of *A. hydrophila* is complex and multifactorial, with the involvement of a number of virulence factors, such as adhesins, toxins and iron acquisition systems^2^. Although new virulence factors are constantly being discovered, the pathogenesis of this bacterium remains unclear.

One of the most fundamental aspects of infectious diseases is microbial access to host nutrients. Bacterial pathogens often encounter a limited availability of carbon, nitrogen, and energy sources during infections while competing with other microorganisms for nutrients^3^. Increasing evidence suggests that pathogens have developed specific metabolic strategies to overcome these restrictions to increase their fitness in the nutrient-poor environments^4^. The role of metabolism is therefore increasingly considered equally important as classical virulence for studying pathogenicity, and metabolic factors required for successful infection are regarded as virulence factors^5^.

Our previous study showed that the virulent *A. hydrophila* strains were equipped with three metabolic pathways for utilizing *myo*-inositol (MI), sialic acid and L-fucose^6^, with the MI metabolic pathway playing a relatively important role in virulence of *A. hydrophila* NJ-35^7^. A number of microorganisms are able to utilize MI as a sole source of carbon and energy, such as *Salmonella enterica* serovar Typhimurium^8^ and *Legionella pneumophila*^9^. In the *A. hydrophila* NJ-35 genome, the inositol catabolic *(iol*) genes are organized in a single cluster with 12 genes that are located on a 32.8-kb genomic island (U876_07765-07910)^6^. The degradation of MI is initiated by 2-dehydrogenase (IolG_1/IolG_2), and enzymes encoded by IolEDBCA are further required for the subsequent degradation steps. The net result of MI metabolism is that MI is converted to a mixture of dihydroxyacetone phosphate and acetyl-CoA. Dihydroxyacetone phosphate can enter the glycolysis metabolic pathway, while acetyl-CoA conveys carbon atoms within acetyl groups to the citric acid cycle to be oxidized for energy production.

IolR is a key player in MI utilization and has been reported to be a negative transcriptional regulator of the entire iol operon in many bacteria, such as *Sinorhizobium meliloti*^10^, *S. enterica*^8^ and *Caulobacter crescentus*^11^. It is possible that the strong repression of *iol* gene transcription is required to maintain a balance between metabolic flexibility and fitness costs^9^. However, the regulatory effects of IolR go beyond MI metabolism. Cordero-Alba et al.^12^ demonstrated that in *S. enterica*, IolR is also involved in the regulation of SrfJ (a *Salmonella* type III secretion system effector). In addition, in *Corynebacterium glutamicum*, IolR was reported to activate the expression of *pck*, which encodes a phosphoenolpyruvate carboxykinase, inhibiting D-xylose uptake^13^. Furthermore, IolR could block the glucose uptake by inhibiting the expression of two glucokinase genes^14–16^. An in vitro study from *S. enterica* has shown that *iolR* directly regulated the expression of reversible lysine acetylation (RLA) system^17^. These studies suggest that *iolR* is not only a transcriptional repressor of MI metabolism but is also involved in the regulation of other genes.

In this study, we explored the function of IolR in *A. hydrophila* NJ-35. We observed that the deletion of *iolR* notably increased the efficiency of inositol utilization in the resulting strain due to significant transcriptional upregulation of the inositol metabolism genes. Interestingly, the loss of *iolR* resulted in a significant increase in autoaggregation and biofilm formation by upregulating the expression of RmpA, a RTX-family protein. To the best of our knowledge.

## Results

### Effect of *iolR* inactivation on bacterial growth

**Fig. 1:**
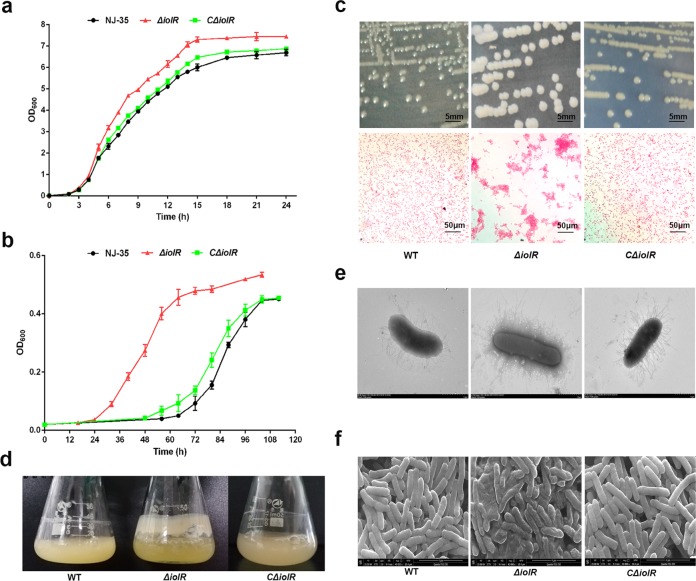
Growth characteristics of the wild-type and *ΔiolR* mutant strains. Growth curve of the wild-type and *ΔiolR* mutant strains in LB medium (**a**) or glucose-free M9 medium supplemented with 50 mM MI (**b**). **c** Comparison of colony and microscopic morphologies of strains on LB plates. The colony was observed after culture for 18 h in LB medium (Scale bar = 5 mm). Simultaneously, the microscopic morphology was observed after Gram staining (Scale bar = 50 μm). **d** Biofilm formation on the flask walls. The bacteria were cultured in LB medium for 18 h at 28 °C. Images were acquired with a camera. **e** TEM images of the wild-type and *ΔiolR* mutant. Scale bar = 1 μm. **f** SEM images of the wild-type and *ΔiolR* mutant. Scale bar = 4 μm.

To evaluate the physiological function and pathogenic significance of the inositol metabolism transcriptional regulator IolR in *A. hydrophila* NJ-35, an *iolR* gene deletion strain was constructed by homologous recombination. We evaluated bacterial growth in LB medium, and as shown in Fig. 1a, the *ΔiolR* had a slightly higher optical density than the wild-type strain; however, both strains entered exponential and stationary phase at the same time indicating no major difference in growth kinetic. This could be due to other carbon sources present in LB broth, such as glucose, that impacted the MI utilization. Therefore, the growth of the *ΔiolR* and wild-type strain was tested in glucose-free M9 medium supplemented with MI as the sole carbon source. As shown in Fig. 1b, the wild-type strain exhibited an approximately 24 h growth delay compared to the *ΔiolR* mutant. Thus, the time required to reach the stationary stage was significantly shortened when the gene *iolR* was inactivated (Fig. 1b), indicating that *iolR* has a negative effect on the inositol utilization efficiency in *A. hydrophila* NJ-35. Furthermore, on LB agar, the *ΔiolR* mutant exhibited a larger colony size compared to the wild-type strain over the same culture period (Fig. 1c). In addition, the *ΔiolR* mutant colony exhibited an off-white color, whereas the wild-type strain and *CΔiolR* strains were primrose yellow (Fig. 1c). At 18 h postculture, bacteria were examined by light microscopy, and while bacterial clumps were observed for the *ΔiolR* mutant, no clumps were observed for the wild-type and *CΔiolR* strains. Interestingly, we noted that large amounts of biofilms were produced by the *ΔiolR* mutant on the flask walls when the bacteria were grown to stationary phase in LB medium (Fig. 1d). Observation by transmission electron microscope (TEM) of *ΔiolR* mutant showed the surface of this bacterial strain was covered by a dense coating of surface-associated structures, whereas no similar structures were found on the surfaces of the wild-type and *CΔiolR* strains (Fig. 1e). In addition, scanning electron microscope (SEM) images revealed that the *ΔiolR* exhibited wrinkle and crumpled morphology, whereas the surface of the wild-type and *CΔiolR* strains appeared smooth with no distinguishing features (Fig. 1f).

### Inactivation of *iolR* leads to a significant transcriptional upregulation of *iol* genes

**Fig. 2:**
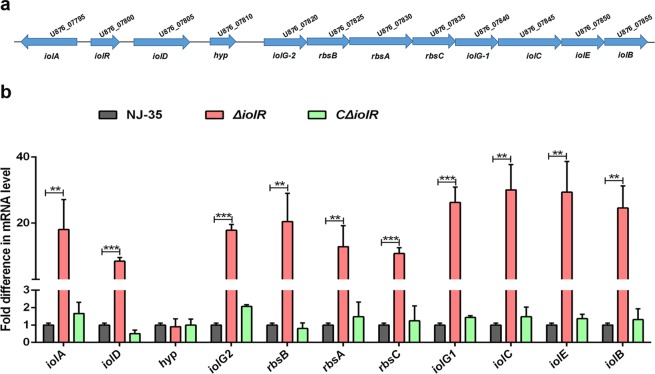
The negative effect of *iolR* on the iol gene cluster of *A. hydrophila* NJ-35. **a** Genetic organization of the *iol* gene cluster of *A. hydrophila* NJ-35. The *iol* gene cluster contains twelve genes predicted to be involved in the metabolism of MI. **b** mRNA expression levels of the *iol* gene cluster after the inactivation of *iolR*. RNA was isolated from the log-phase bacteria that had been cultured in LB broth, and then converted to cDNA. qRT-PCR was performed, and the data were normalized to expression of the internal housekeeping gene *recA*. The results are expressed as *n*-fold increases with respect to the control. Error bars represent the standard deviations from at least three independent experiments. Statistical significance was determined using a two-tailed unpaired Student’s *t* test. **P < 0.01, ***P < 0.001.

Considering that the *ΔiolR* mutant had higher inositol utilization efficiency than the wild-type strain, we further examined the transcriptional levels of the *iol* genes in the *ΔiolR* strain. The deletion of *iolR* resulted in a significant upregulation of the *iol* gene transcription levels, except for a gene encoding for a hypothetical protein (*hyp*), while the transcriptional levels of the *iol* genes in the *CΔiolR* strain were restored to the wild-type levels (Fig. 2). The data suggest *iolR* is responsible for the negative regulation of *iol* gene transcription.

### IolR is involved in bacterial autoaggregation

**Fig. 3:**
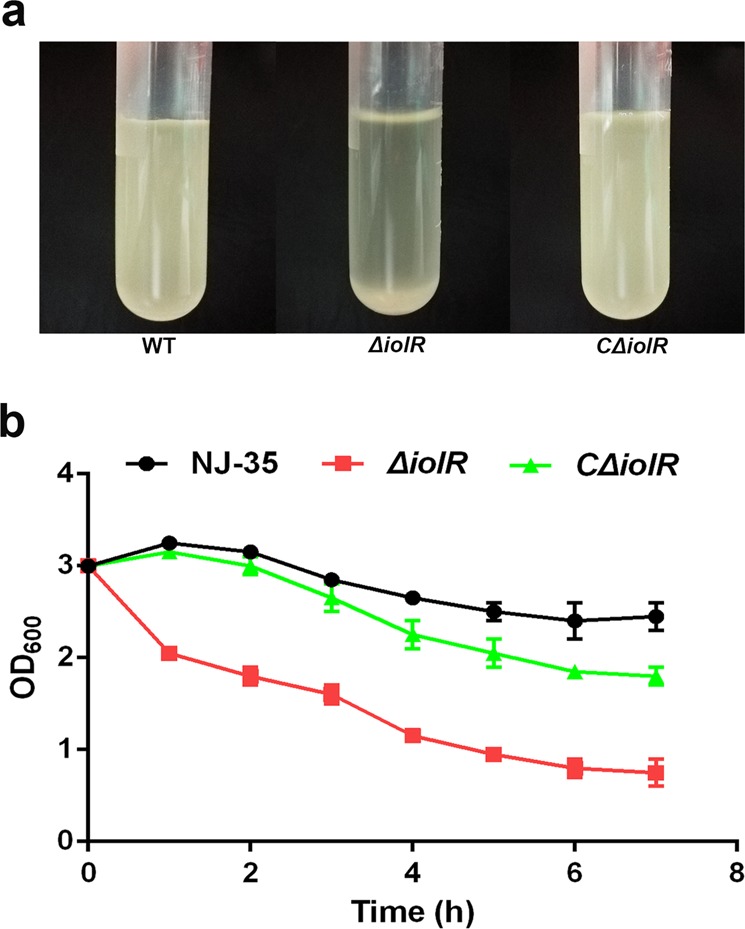
The *iolR* mediates *A. hydrophila* autoaggregation. **a** Autoaggregation of the wild-type and *ΔiolR* mutant strains was measured macroscopically in stationary tubes after culturing for 7 h. **b** Sedimentation kinetics of the wild-type and *ΔiolR* mutant strains. The bacteria were grown in LB medium for 18 h and adjusted to an OD_600_ of 3.0, after which they were grown under static conditions. The OD_600_ of the upper layer of the culture was measured every 1 h. Error bars represent the standard deviations from at least three independent experiments.

As shown by Fig. 1c, the *ΔiolR* mutant exhibited dense bacterial aggregates in light microscopy observations, suggesting a possible role for IolR in autoaggregation. To evaluate bacterial autoaggregation, we performed sedimentation assays by measuring the OD_600_ value of the bacterial culture supernatant^18^. After stationary cultures were grown for 7 h, most *ΔiolR* cells settled to the bottom of the tube, and the supernatant became transparent, whereas the cultures of the wild-type and *CΔiolR* strains remained turbid (Fig. 3a). We next compared the sedimentation kinetics of the wild-type and *ΔiolR* strains. As shown in Fig. 3b, in the strain deleted for *iolR*, a rapid decrease in the OD_600_ value was observed during the static growth. After 7 h, the maximal autoaggregation of the *ΔiolR* strain was reduced 69.4%, as evaluated by the OD_600_ value of the culture supernatant, compared to the wild-type strain. Complementation of *iolR* gene resulted in recovery of its autoaggregation properties. Together, these results suggest that IolR is involved in *A. hydrophila* autoaggregation.

### Ca^2+^ is required for *iolR-*induced autoaggregation

**Fig. 4:**
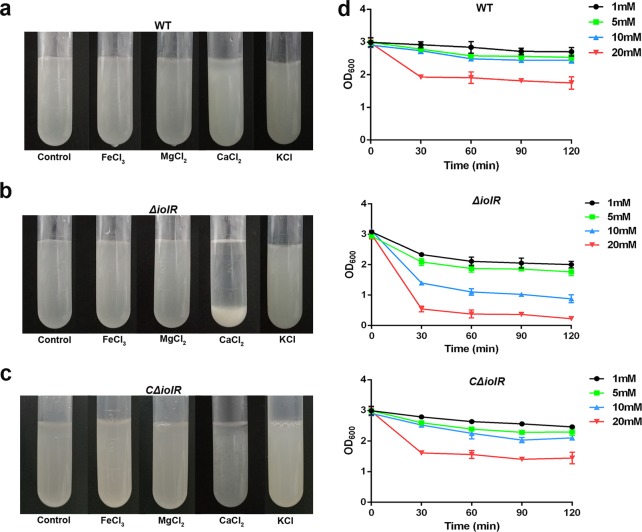
Ca^2+^ is required for *iolR*-dependent autoaggregation. Autoaggregation of WT (**a**), *ΔiolR* (**b**) and *CΔiolR* (**c**) was measured macroscopically in stationary tubes after culturing for 2 h. Bacteria were grown in LB medium for 18 h, then harvested by centrifugation at 2000 × *g* for 10 min, and resuspended in PBS supplemented with 10 mM FeCl_3_, CaCl_2_, MgCl_2_ or KCl. Images of autoaggregation were obtained using a camera after a 2-h incubation. **d** Sedimentation kinetics of WT, *ΔiolR* and *CΔiolR* with 1 mM, 5 mM, 10 mM, and 20 mM CaCl_2._ The OD_600_ of the upper layer of the culture was measured every 30 min. Error bars represent the standard deviations from at least three independent experiments.

Metal cations were previously reported to be involved in bacterial autoaggregation^18,19^. To determine whether *iolR-*induced autoaggregation was related to the presence of metal cations, we measured bacterial sedimentation with 10 mM FeCl_3_, MgCl_2_, CaCl_2_ or KCl in phosphate-buffered saline (PBS). For the wild-type strain, no significant sedimentation was observed when the buffer was supplemented with a metal chloride (Fig. 4a). However, rapid sedimentation of the *ΔiolR* strain occurred with the addition of CaCl_2_, whereas no sedimentation was observed in the presence of the other three chlorides (Fig. 4b). The autoaggregation characteristics were restored in the *CΔiolR* strain (Fig. 4c). These data indicate that *iolR-*induced autoaggregation is related to the presence of Ca^2+^, and chloride ions have no effect on the autoaggregation of *A. hydrophila*. To determine whether the concentrations of Ca^2+^ affected the sedimentation of the *ΔiolR* mutant, we measured the sedimentation kinetics of *A. hydrophila* strains in PBS supplemented with 1, 5, 10, and 20 mM CaCl_2_, and the OD_600_ of the bacterial suspensions was monitored for 2 h. The data showed that Ca^2+^ could increase the rate of sedimentation of *A. hydrophila* strains in a dose-dependent manner. After 120 min, the maximal autoaggregation of the *ΔiolR* strain was reduced 88.9% compared to the wild-type strain in the presence of 20 mM CaCl_2_, suggesting that the *ΔiolR* mutant was more sensitive to Ca^2+^ than the wild-type strain (Fig. 4d).

### Ca^2+^-mediated sedimentation of the *ΔiolR* is likely related to the increased expression of surface proteins

**Fig. 5:**
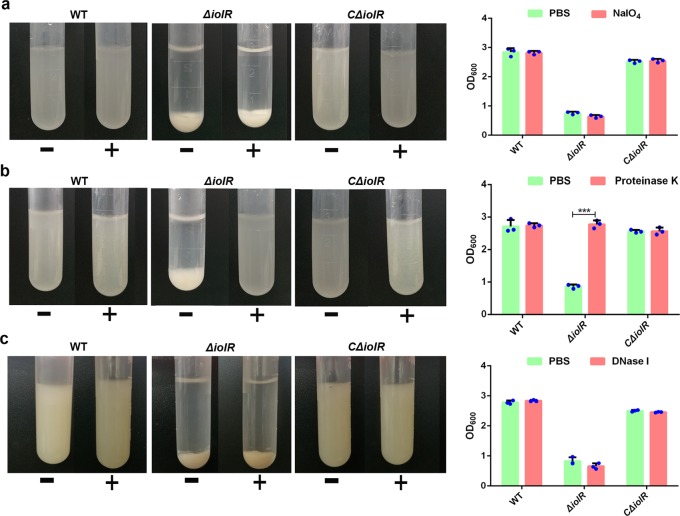
Effects of NaIO_4_, proteinase K and DNase I on cellular autoaggregation. Bacteria cultured for 18 h were washed with PBS and adjusted to an OD_600_ of 3.0. The cells were then treated with NaIO_4_ (**a**), proteinase K (**b**) or DNase I (**c**) for 2 h at 28 °C. Cells were subsequently harvested and resuspended in PBS, after which 10 mM CaCl_2_ was added and the cultures were incubated at 28 °C for 2 h. “+“ indicates treatment with NaIO_4_, proteinase K or DNase I. “−” indicates treatment with PBS. The OD_600_ of the upper layer of the culture was measured at the same time point. Error bars represent the standard deviations from at least three independent experiments. Statistical significance was determined using a two-tailed unpaired Student’s *t* test. ***P < 0.001.

Proteins, polysaccharides and eDNA have been reported to play an important role in interbacterial and bacterial-surface interactions^18,20,21^. To investigate the possible mechanism of bacterial sedimentation, we treated bacterial cultures with proteinase K, NaIO_4_ or DNase I. After treating bacterial-surface polysaccharides with NaIO_4_ or DNase I, the *ΔiolR* mutant still exhibited significant autoaggregation in PBS containing Ca^2+^, as observed by visual inspection of the tubes (Fig. 5a, c). However, the *ΔiolR* strain was observed to have lost its sensitivity to Ca^2+^ after bacterial-surface proteins were treated with proteinase K (Fig. 5b).

### Inactivation of *iolR* increases biofilm formation

**Fig. 6:**
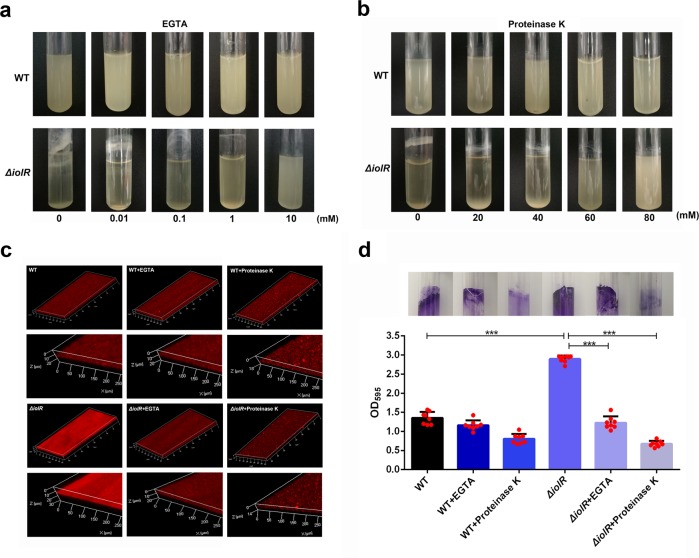
Biofilm determination. Effect of EGTA (**a**) and proteinase K (**b**) on biofilm formation of the wild-type and *ΔiolR* mutant strain. The bacteria were cultured in LB medium supplemented EGTA at a range of concentrations from 0.01 to 10 mM or proteinase K at a range of concentrations from 20 to 80 mM for 18 h at 28 °C. Images were acquired with a camera. **c** CLSM images of biofilms of the wild-type and *ΔiolR* mutant strains. The viable cells exhibit red fluorescence. **d** Biofilm formation was observed by crystal violet staining and quantification analysis was performed by measuring the OD_595_ values. Error bars represent the standard deviations from at least three independent experiments. Statistical significance was determined using a two-tailed unpaired Student’s *t* test. ***P < 0.001.

As bacterial autoaggregation is a key step in biofilm formation^22^, we investigated the role of IolR in biofilm formation. As shown in Fig. 6a, biofilm formation of *ΔiolR* mutant showed a strong increase compared to the wild-type strain in non-supplemented medium. In addition, a negative correlation between the Ca^2+^ concentration and biofilm formation was observed by the addition of calcium chelator EGTA. The amount of biofilm formed was decreased with the increasing of EGTA concentration. Similarly, the addition of proteinase K, which acts as an extracellular protein-degrading enzyme, inhibited the accumulation of biofilm in *ΔiolR* mutant. Confocal laser scanning microscopy (CLSM) analysis showed that the *ΔiolR* mutant exhibited a drastically enhanced ability to form biofilm. And the biofilm-formation ability of *ΔiolR* was decreased in the presence of 10 mM EGTA. Similarly, the addition of 80 mM proteinase K caused a serious decrease in biofilm formation (Fig. 6c). Consistent with the CLSM result, quantification of the biofilm biomass by crystal violet staining showed a 2.14-fold increase in the *ΔiolR* compared with the wild-type strain, but a significant decrease when adding EGTA or proteinase K (Fig. 6d). These results suggested that Ca^2+^ and surface proteins are necessary for the increased biofilm biomass caused by the inactivation of *iolR*.

### Comparative transcriptomics analysis

**Fig. 7:**
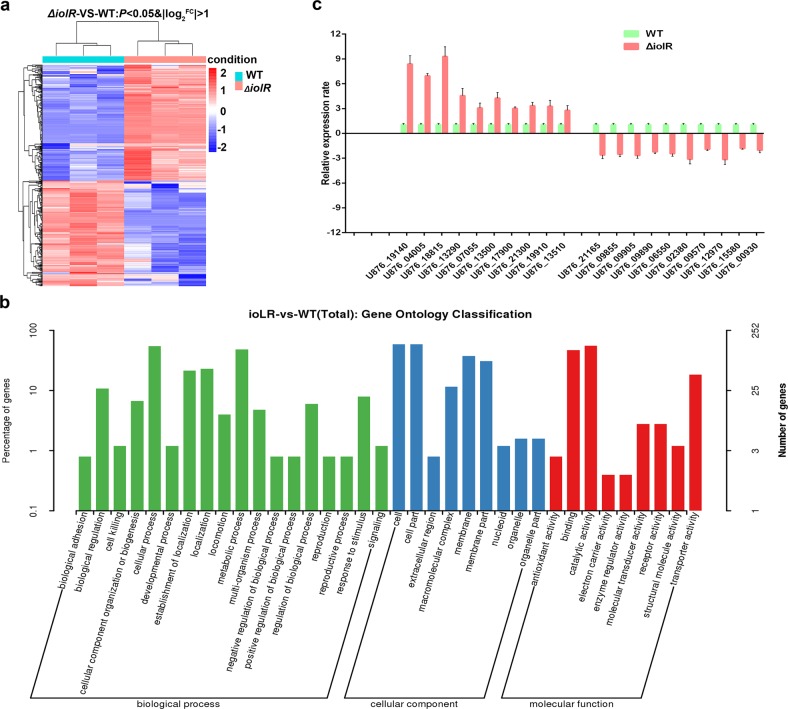
Comparative transcriptomics analysis. **a** Heat map of the 447 identified genes. Red font indicate upregulated expression genes and blue font indicate downregulated expression genes in *ΔiolR* mutant, respectively. **b** Gene Ontology (GO) classification of differentially expressed genes. The differentially expressed genes are grouped into three hierarchically structured terms: biological process, cellular component and molecular function. **c** Relative mRNA expression levels of 20 differentially expression genes in *ΔiolR* mutant and wild-type strains. The results were normalized to those of RecA and then were expressed as n-fold increases with respect to the control. Error bars represent the standard deviations from at least three independent experiments.

For a comprehensive analysis of the function of the transcriptional suppressor IolR, we investigated the differential transcriptome profiles of the wild-type and *iolR* mutant strains. A total of 447 genes were differentially expressed (change>2.0 fold) in the *ΔiolR*, including 234 upregulated and 213 downregulated genes. Figure 7a shows the hierarchical clustering of the identified genes, where an increasing color intensity indicates increasing gene expression levels. Further, we classified the 447 differential proteins by Gene Ontology (GO) categories. As shown in Fig. 7b, these differentially expressed genes are involved in membrane part, transportation, cellular metabolism and signal transduction. Then quantitative PCR of 20 genes, including 10 upregulated and 10 downregulated genes, was conducted to validate that the gene expression patterns were consistent with the varying patterns of the transcriptomic data (Fig. 7c).

### U876_04005 is required for the autoaggregation caused by *iolR* deletion

**Fig. 8:**
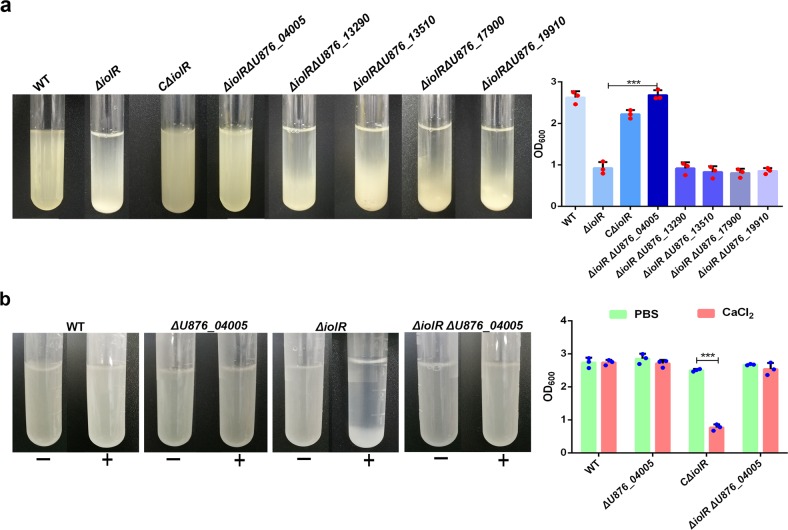
U876_04005 is required for *iolR* mediated aggregation. **a** Aggregation of *ΔiolRΔU876_13290*, *ΔiolRΔU876_13510*, *ΔiolRΔU876_17900*, *ΔiolRΔU876_19910* and *ΔiolRΔU876_04005* was measured macroscopically in stationary tubes after culturing for 7 h. **b** Ca^+^-mediated aggregation disappeared in *ΔiolRΔU876_04005*. Bacteria were grown in LB medium for 18 h, harvested by centrifugation, and then resuspended in PBS supplemented with 10 mM CaCl_2_. Images of autoaggregation were obtained using a camera after a 2-h incubation. The OD_600_ of the upper layer of the culture was measured at the same time point. Error bars represent the standard deviations from at least three independent experiments. Statistical significance was determined using a two-tailed unpaired Student’s *t* test. ***P < 0.001.

Based on the transcriptomic data, we identified five surface proteins (U876_04005, U876_13290, U876_13510, U876_17900 and U876_19910) whose expressions were upregulated in the *iolR* mutant. To further investigate whether the autoaggregation phenotype observed with *ΔiolR* mutant was associated with these surface proteins, we constructed the single gene mutant in *ΔiolR* background. Sedimentation assay was performed to evaluate bacterial autoaggregation. As shown in Fig. 8, *ΔiolRΔU876_13290*, *ΔiolRΔU876_13510*, *ΔiolRΔU876_17900* and *ΔiolRΔU876_19910* could still form autoaggregates as observed in *ΔiolR*, whereas the phenomenon of autoaggregation disappeared in *ΔiolRΔU876_04005*. These data suggest that U876_04005 is the IolR-regulated target gene involved in the formation of aggregates. In addition, to determine whether U876_04005-mediated autoaggregation is calcium dependent, we assessed the sedimentation of *ΔiolRΔU876_04005* in PBS buffer supplemented with 10 mM CaCl_2_. The autoaggregation did not occur in *ΔiolRΔU876_04005*, indicating calcium-induced autoaggregation requires U876_04005.

### U876_04005 is required for the increased biofilm formation caused by *iolR* deletion

**Fig. 9:**
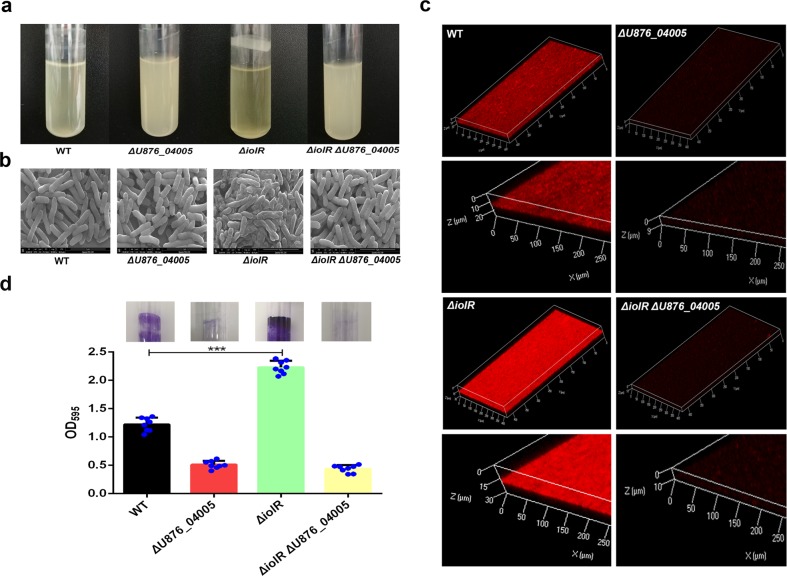
Biofilm formation of *ΔU876_04005* and *ΔiolRΔU876_04005*. **a** Biofilm formation of the wild-type and *U876_04005* mutants on the tube wall. **b** The morphology of the wild-type and *U876_04005* mutants using SEM. **c** Bacterial biofilm of the wild-type and *U876_04005* mutants using CLSM. **d** Quantitation of biofilm. The optical density at OD_595_ was recorded. Error bars represent the standard deviations from at least three independent experiments. Statistical significance was determined using a two-tailed unpaired Student’s *t* test. ***P < 0.001.

As shown in Fig. 9a, the biofilms produced by *ΔiolRΔU876_04005* on the tube wall were significantly reduced compared to the *ΔiolR*. Bacterial morphology observed by SEM was shown in Fig. 9b. The surface of *ΔiolRΔU876_04005* was smooth and clear like the wild-type strain. Compared to the *ΔiolR*, biofilms of *ΔiolRΔU876_04005*, as observed by CLSM, showed significant decrease in thickness (Fig. 9c), and quantification by crystal violet staining displayed a 69.31% reduction in the biomass (Fig. 9d). These results demonstrated that *U876_04005* might be involved in the increased biofilm formation caused by the *iolR* deletion.

### The repressor IolR binds to the promoter of *rmpA*

**Fig. 10:**
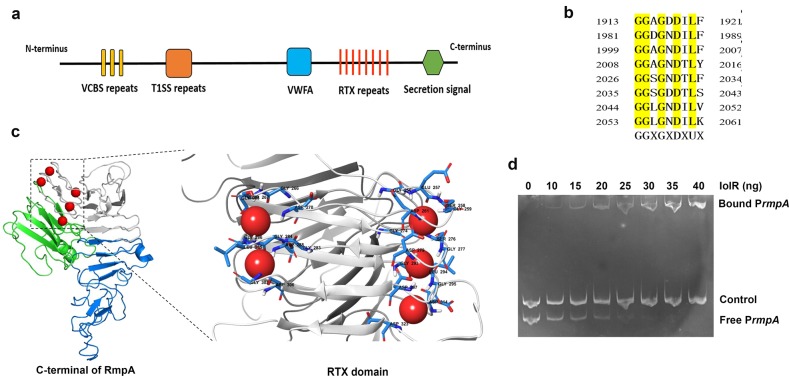
IolR directly regulated the expression of RmpA. **a** Schematic representation of RmpA. VCBS is shown as yellow boxes. The VWFA domain, RTX repeats and type I secretion target signal are shown as a blue box, vertical red lines and green hexagon, respectively. **b** Individual RTX nonapeptide repeats of RmpA after their alignment. The nonapetide consensus sequence, G-G-X-G-X-(D/N)-X-U-X, is shown below, where X means any amino acid and U means a hydrophobic amino acid. **c** The 3D structure of RTX domain at the C-terminal of RmpA (residues 1762 to 2178). Calcium ions are shown as red spheres which bound in the turns between two β strands. **d** EMSA showing IolR binding to the promoter region of RmpA. Promoter DNA was incubated with or without increasing amounts of purified IolR. A 400 bp sequence of the *ΔU876_13290* promoter served as a negative control.

BLASTn based searches and sequence analyses revealed that U876_04005 encodes a very large protein, with a hypothetical molecular mass of 223 kDa. This newly identified protein was designated as RmpA (accession number: AKJ33327). Amino acid sequence analysis revealed that RmpA is composed of the VCBS domain (504-602aa), vWFA domain (1631-1761aa), RTX motif (1913-2061aa) and type I secretion C-terminal target domain (2108-2175aa) (Fig. 10a). VCBS domain are found in many large proteins annotated as adhesins or biofilm-associated proteins and predicted to be associated with bacterial adherence^23^. vWFA domain is involved in cell-cell contact and is widely conserved in prokaryotes^24^. RTX motif comprises eight glycine-rich and aspartate-containing nonapeptide repeats of the consensus sequence G-G-X-G-X-(D/N)-X-U-X, where X is any amino acid, and U is a conserved hydrophobic residue (Fig. 10b). These repeats form a parallel β-roll motif which serves to bind calcium. The presence of calcium binding sites was further supported by *in silico* modeling of the three-dimensional structure of the region spanning the 400 C-terminal amino acids of RmpA. As shown in Fig. 10d, there are five putative Ca^2+^-binding sites located in the RTX motif. Also, the type I secretion C-terminal target domain in RmpA indicated that this protein might be secreted by type I secretion system. These characteristic domains imply that RmpA may be a putative RTX-family adhesin.

Based on the transcriptional analysis described above (Fig. 7), the expression of *rmpA* was increased when *iolR* was deleted. To test whether IolR acts as a repressor to regulate the expression of *rmpA* directly, we performed an electrophoretic mobility shift assay (EMSA) with DNA fragments containing the putative promoter of *rmpA*. The EMSA result showed that IolR was able to bind the promoter region of *rmpA*, and displayed a dose-dependent mobility shift.

## Discussion

Previous reports indicated that the *iolR* gene product is involved in the regulation of the myo-inositol catabolism in many bacteria^10,25^. In this study, it is also found that the absence of *iolR* shortened the *A. hydrophila* growth retardation in medium with inositol as a sole carbon source. Further examination showed a significant upregulation in transcriptional levels of MI metabolic genes resulting from the *iolR* deletion. The data indicated that IolR is a negative regulator of the MI metabolic pathway. Notably, the *iolR* mutant showed significantly different growth characteristics from the wild-type strain in LB medium. In standing cultures of the *iolR* mutant, cells of this strain quickly aggregated and settled to the bottom of the tubes. The ability of the *ΔiolR* mutant to aggregate and settle may provide more opportunities for bacteria to adhere and invade host cells. *Legionella* collagen-like protein (Lcl)-dependent autoaggregation has been reported to potentiate the infection of *Acanthamoeba castellanii* by *Legionella pneumophila*^18^. Similarly, the aggregative state increases adherent-invasive *Escherichia coli* susceptibility to phagocytosis by macrophages and subsequent intracellular survival^26^.

The reason for the autoaggregation of the *iolR* mutant is not clear. According to our results, IolR mediates the autoaggregation through a Ca^2+^-dependent mechanism. In the presence of Ca^2+^, the absence of *iolR* leads to an increase in cellular autoaggregation and sedimentation, which increased as concentration of Ca^2+^ increased. This finding is consistent with a previous study, which reported that *L. pneumophila* is capable of autoaggregating in a process mediated by Lcl in a divalent-cation-dependent manner^18^. This supports the idea that enhanced sedimentation might be due to the increased binding of Ca^2+^. Calcium binding proteins have been previously shown to influence the biofilm formation of different bacterial species. A recent works on *Vibrio vulnificus* demonstrated that calcium binding induces conformational changes of CabA to multimeric forms, and together with exopolysaccharides, assemble a functional matrix which is essential for the development of the biofilm structure^27^. In *Pseudomonas putida*, Ca^2+^ facilitates the clustering of LapF to form fibrillar structure and thus promotes aggregations of adjacent cells, which lead to microcolony formation and subsequent biofilm maturation^28^. Autoaggregation and biofilm are often associated phenotypes. The sedimentation is usually generated by interactions mediated by aggregation factors involved in biofilm formation^29^. In this work, we demonstrated that the deletion of *iolR* caused a significant increase in biofilm formation. It would be interesting to know if, as in the case of IolR, Ca^2+^ binding affects the function of bacterial-surface molecules in autoaggregation and biofilm formation.

To determine the factors affecting the sedimentation characteristics of the strains, we removed proteins, polysaccharides or eDNA on the bacterial surface using proteinase K, NaIO_4_ or DNase I and then examined the bacterial settlement in the presence of Ca^2+^. The results showed that the sensitivity of the *iolR* mutant to Ca^2+^ may be related to the increased expression of surface proteins. To identify the surface proteins possibly regulated by the *iolR*, we investigated the differential transcription profiles of the wild-type and *iolR* mutant strains using comparative transcriptome method. Upregulation of RmpA, encoded by the locus tag U876_04005, in the *iolR* mutant attracted our attention. RmpA belongs to RTX protein family which has been reported to be involved in aggregation and biofilm formation^30^. In *Shewanella oneidensis*, autoaggregation and increased biofilm formation at the stages of both initial surface attachment and biofilm maturation, require upregulation of the RTX protein BpfA^31^. Similarly, the RTX adhesion LapA produced by *Pseudomonas fluorescens* contributes to the early step of biofilm formation and the development of mature biofilm structure^32^. To substantiate the role of *rmpA* in biofilm formation, we deleted *rmpA* in *ΔiolR* background and found that the biofilms were reduced in *ΔiolRΔrmpA* mutant. We further demonstrated that calcium-mediated aggregation disappeared as well in *ΔiolRΔrmpA* mutant. These data indicated the strong involvement of the upregulated *rmpA* gene in *ΔiolR* aggregation, and the necessity of the RmpA surface protein to the *A. hydrophila* phenotype with its increased biofilm formation. We can not determine whether the aggregation/biofilm phenotype of the IolR mutant would be exclusively due to the upregulation of the RpmA protein whereas modification of the other genes in the IolR regulon would not be related with the phenotype. We will further evaluate this in the future study.

Contrary to our findings, an earlier report in *Staphylococcus aureus* showed that calcium inhibited the large surface protein Bap-mediated biofilm formation, and this inhibition effect might be due to a conformational change in Bap that affects its ability to form biofilms^33^. We speculate that the influence of calcium on different large surface proteins may be associated with different Ca^2+^ binding sites. It is worth noting that the C-terminal RTX domain of RmpA does not correspond to EF-hand-like motifs in Bap. Instead, the Ca^2+^ binding sites in RmpA resemble those decribed in BrtA, a RTX family adhesin which has been shown to be involved in *Bordetella bronchiseptica* biofilm formation in a calcium-dependent way^23^. Bumba et al.^34^ have reported that the RTX protein family requires calcium to exert the biological functions. This led us to speculate that the interaction of RmpA with Ca^2+^ might change the protein conformation and/or the interaction between RmpA proteins of adjacent cells, thus causing autoaggregation and accelerated biofilm formation. To address this, we tried to analyze the RmpA conformation by western-blot in native gel with bacteria grown in the presence or absence of calcium. But calcium has no effects on the migratelength of RmpA (Supplementary Fig. 1). We can not rule out the possibility that calcium binding might trigger the folding of RTX domain (fold into an idiosyncratic parallel β-helix structure), but did not alter the overall structure of RmpA. Similar phenomenon has been described in *S. enterica*, where the incubation of RTX biofilm-associated protein SiiE with Ca^2+^ had only little effect on the curvature of the full-length SiiE molecule^35^. Maybe several biophysical methods such as circular dichroism, fluorescence, infrared, Raman and NMR spectroscopies, will help to clarify the mechanism of the interaction of RmpA with Ca^2+^.

In conclusion, this study confirms that IolR is involved in downregulating the aggregative phenotype and biofilm formation in *A. hydrophila* by downregulating the expression of RmpA, which further decreases the chances of contact with calcium and thus restrains the cell-cell interactions needed for agglutinates formation and the development of biofilm. This study provides insights into the role of IolR in *A. hydrophila*, and sheds new light on the target for treating this bacterial infection.

## Methods

### Bacterial strains and culture conditions

The bacterial strains and plasmids used in this study are listed in Supplementary Table 1. *A. hydrophila* NJ-35 (accession number CP006870), which belongs to the ST251 clonal group, was isolated from dead cultured cyprinoid fish in the Jiangsu province of China in 2010^6^.

*A. hydrophila* and *E. coli* strains were grown in Luria-Bertani broth (LB) medium. For solid media, 1.5% agar (wt/vol) was added. When necessary, the medium was supplemented with the following antibiotics: chloramphenicol (Cm) (Sigma, St. Louis, USA), 34 µg/mL for *E. coli*; ampicillin (Amp) (Sigma), 100 µg/mL for *A. hydrophila*. The *A. hydrophila* and *E. coli* strains were grown at 28 and 37 °C, respectively.

### Construction of gene deletion mutants

The *iolR* gene inactivation was performed via homologous recombination using the suicide plasmid pYAK1. First, the upstream and downstream flanking regions of the *iolR* gene were PCR-amplified using two sets of primers, *iolR*-1/*iolR*-2 and *iolR*-3/*iolR*-4 (Supplementary Table 2), and the flanking regions were ligated using the primer pair *iolR*-1/*iolR*-4. The fusion fragment was cloned into the pYAK1 suicide plasmid with the restriction enzyme site *Bam*HI. The recombinant plasmid was transformed into *E. coli* SM10 competent cells. The donor strain *E. coli* SM10-pYAK1 and the recipient strain *A. hydrophila* NJ-35were cultured in LB broth without antibiotics until log phase was reached. Cells were mixed at a ratio of two-to-one vol/vol in medium, spotted on a nylon filter on an LB plate and conjugated for 12 h at 28 °C. Then the recombination vector was conjugated into the wild-type strain, and transconjugants were selected by plating on LB agar containing Amp (100 µg/mL) and Cm (34 µg/mL) and were confirmed by PCR. Next, colonies (Amp and Cm) were inoculated in LB broth supplemented with 20% sucrose to induce a second crossover event. The suspected mutants were verified by PCR. Using the same approach, additional deletion mutants were also constructed.

### Construction of the complementation strain

The complementation of the *ΔiolR* mutant strain was performed using the pMMB207 shuttle plasmid. A DNA fragment, including the *iolR* gene and its putative promoter, was amplified using the primer pair *iolR*-C-F/R and ligated into pMMB207. The recombinant plasmid was transferred into *E. coli* SM10 competent cells by chemical transformation and then conjugated into the mutant strain *ΔiolR*. In addition, the pMMB207 empty plasmid was also transformed into the wild-type strain to serve as controls. PCR amplification was performed to verify the generation of the complemented strain.

### Growth assay

A single colony of each strain was cultured in LB medium at 28 °C for 8 h, after which the cells were transferred to fresh LB medium and cultured to the logarithmic-phase (OD_600_ = 0.6). Cells were pelleted by centrifugation at 5000 × *g* for 10 min, washed three times and later resuspended in PBS, and then 200-µL aliquots of the suspension were transferred into Erlenmeyer flasks containing 20 mL of LB or glucose-free M9 medium supplemented with 50 mM MI (Aladdin, Shanghai, China). Next, the Erlenmeyer flasks were incubated at 28 °C with shaking. Bacterial growth was examined at regular intervals by monitoring the OD_600_ using a spectrophotometer (BIO-RAD, USA). The assay was performed in three independent experiments.

### Autoaggregation assay

Bacteria were grown in LB medium for 18 h at 28 °C and washed three times with LB, then adjusted to an OD_600_ of 3.0 in a new tube with the final volume of 3 mL. To evaluate autoaggregation, the OD_600_ value of the culture supernatant was measured every hour using a spectrophotometer.

In addition, to investigate the role of metal cations in autoaggregation, bacteria cultured for 18 h were pelleted by centrifugation at 2000 × *g* for 10 min at 4 °C, washed three times and then resuspended in PBS supplemented with 10 mM FeCl_3_, CaCl_2_, MgCl_2_ or KCl. Images of *A. hydrophila* autoaggregation in tubes were obtained using a camera (Nikon D7100, Japan) after a 2-h incubation.

### Transmission electron microscopy

*A. hydrophila* strains were grown for 16 h in tubes. Bacterial suspensions were adsorbed on formvar-carbon coated copper grids (300mesh) for 5 min. After that, the grids were washed three times on droplets of distilled water and fixed with glutaraldehyde prepared in 0.1 M sodium cacodylate buffer. The grids were negatively stained with 1% sodium-phosphotungstic acid (pH 7.2) for 5 min, and excess solution was removed. All samples were observed on a Hitachi 600 transmission electron microscope.

### Scanning electron microscope

The bacteria were grown to stationary phase, centrifuged at 4000 × *g* for 5 min and then washed three times with PBS. The bacterial suspensions were adjusted to an OD_600_ of 1.0 using a spectrophotometer. Cells were pelleted by centrifugation at 4000 × *g* for 10 min, and then fixed by 2.5% glutaraldehyde for 3 h. Next, the specimens were dehydrated with a water/ethanol gradient of 30%, 50%, 70%, 95% and 100% ethanol, then transferred to absolute acetone for 30 min and dried at room temperature. Samples were coated with gold film by sputter coating and viewed using a FEI Quanta FEG250 SEM.

### Effects of surface proteins, polysaccharides and eDNA on Ca^2+^-mediated autoaggregation

Bacteria were grown in LB medium for 18 h at 28 °C and then were washed three times with PBS and adjusted to an OD_600_ of 3.0. The bacteria were treated with proteinase K (0.5 mg/mL), NaIO_4_ (2.14 mg/mL) or DNase I (1 mg/mL) for 2 h at 28 °C. Cells were harvested by centrifugation at 2000 × *g* for 10 min, washed three times and then resuspended in PBS. Next, CaCl_2_ was added to the culture at a final concentration of 10 mM and the cultures were incubated at 28 °C for 2 h. The tubes were photographed to document the different settling patterns.

### Comparative transcriptome analysis

Bacteria were grown in LB medium for 18 h at 28 °C. Total RNA was purified using an RNAqueous kit (Thermo Fisher Scientific, San Jose, CA, USA) according to the manufacturer’s instructions. The mRNA was enriched using a MICROBExpress Kit (Ambion, USA), and determined on Agilent 2100 Bioanalyzer. Paired-end sequencing was processed by the Hiseq™2000 (Illumina, San Diego, CA, USA) sequencer. The assembled reads were mapped to the complete genome of the *A. hydrophila* NJ-35 strain (http://www.ncbi.nlm.nih.gov/nuccore/ CP006870.1). The differentially expressed genes were characterized by GO categories, KEGG enrichment, and clustering analyses.

### Quantitative reverse transcription-PCR (qRT-PCR)

To validate the transcriptomics results, we used qRT-PCR to measure the transcription levels of randomly selected genes. The primer pairs used are shown in Supplementary Table 2. Total RNA was extracted from stationary-phase bacteria using an E.Z.N.A. bacterial RNA isolation kit (Omega, Beijing, China). cDNA synthesis was performed using HiScript II QRT Supermix (Vazyme, Nanjing, China). The mRNA transcription levels of the 20 genes were examined individually using a One Step qRT-PCR SYBR Green kit (Vazyme Biotech) in an ABI PRISM 7300 Fast Real-time PCR machine. The housekeeping gene *recA* was chosen as an internal control for qRT-PCR, and the 2^−∆∆Ct^ method was used to calculate the fold-change of mRNA expression levels.

### Electrophoretic mobility shift assay

To detect if IolR could bind to the promoter DNA of RmpA, EMSA was performed using an EMSA kit. Briefly, the 300-bp promoter region of *rmpA* was amplified using a pair of specific primers and purified using a gel extraction kit. The purified PCR product (40 ng) was incubated with the increasing amounts of purified IolR-His fusion protein (0-40 ng) in 20-μL volume of binding buffer (50 mM Tris-HCl, 50 mM KCl, 5 mM MgCl_2_, 1 mM EDTA, and 10% glycerol) for 50 min at 16 °C. After the reaction, 4 μL of DNA loading buffer was added to stop the EMSA reaction and the reaction mixtures were loaded into a 6% polyacrylamide gel. Electrophoresis was performed under 200 V for about 60 min using 0.5 × TBE buffer (44.5 mM Tris, 44.5 mM boric acid, 1 mM EDTA, pH 8.0). The gel was stained in 0.5×TBE buffer containing 1 × SYBR Gold nucleic acid staining solution for 10 min, and then the image was recorded.

### Biofilm-formation assay

CLSM was performed to analyze the three-dimensional architecture of biofilms. Briefly, the stationary-phase bacterial cultures were adjusted to an OD_600_ of 1.0 and then diluted 1:1000 in LB medium. Two milliliters of these dilutions were dispensed into 6-well polystyrene plates containing pre-sterilized microscopic glass slides for biofilm growth. Then the plates were incubated at 28 °C for 24 h without agitation, and the glass slides were carefully washed three times with sterile PBS. Next, propidiumiodide (PI, Sigma, Louis, MO, USA) was added to each glass slide with a final concentration of 100 µg/mL for 30 min. Then, the slides were washed five times with PBS to remove the unbound dye. The biofilms grown on the slides were observed by CLSM (Carl Zeiss LSM700, Oberkochen, Germany) using an argon laser. Seven random spots were measured for each of the three replicate glass slides.

Biofilm formation was also quantitatively measured by crystal violet staining. Briefly, Single colonies of *A. hydrophila* strains were inoculated into LB medium and cultured at 28 °C for 8 h. After being adjusted to an OD_600_ of 0.1, the cell suspensions were subcultured at a 1:200 dilution in glass tubes in a final volume of 3 mL LB broth, followed by an incubation with shaking for 18 h at 180 rpm at 28 °C °C. Fresh LB medium was added to tubes as a blank control. Next, the medium was decanted, and the tubes were washed three times with sterile PBS. After washing, the bacterial cells were fixed with 200 µL of methanol for 15 min and allowed to air dry at room temperature. After drying, 200 μL of a 1% crystal violet solution (wt/vol) was added to each tube, and the cells were stained for 10 min at room temperature. The tubes were then washed with ddH_2_O to remove unbound crystal violet. Subsequently, the bound crystal violet was solubilized using 95% ethanol for 10 min, and the optical density was measured at OD_595_. The assay was performed in three independent experiments.

### Statistical analyses

The data obtained in this study were statistically analyzed using the SPSS software (SPSS 16 for Windows, SPSS Inc., Chicago, IL, USA). All statistical analyses were performed using unpaired two-tailed Student’s *t* tests. *P* values < 0.05 were considered to be statistically significant. All data are presented as the means ± standard deviations derived from a minimum of three biological replicates.

### Reporting summary

Further information on research design is available in the Nature Research Reporting Summary linked to this article.

## Supplementary information

Supplementary Information

Reporting Summary

## Data Availability

The data that support the findings of this study are available from the corresponding author upon reasonable request. Supplementary information is available at npj Biofilms and Microbiomes’s website. The RNA-Seq data generated from this study were submitted to the NCBI Sequence Read Archive (SRA) under accession numbers SRR11470407 to SRR11470412.
