# Supplementary material for: IolR, a negative regulator of the myo-inositol metabolic pathway, inhibits cell autoaggregation and biofilm formation by downregulating RpmA in Aeromonas hydrophila
Source: NPJ Biofilms Microbiomes. 2020 May 20;6:22. doi: 10.1038/s41522-020-0132-3 (PMC7239862; doi:10.1038/s41522-020-0132-3)
Supplement: Supplementary file 1 — Supplementary Information [file 41522_2020_132_MOESM1_ESM.pdf]

Supplementary Table 1 Bacterial strains and plasmids used in this study

Strain or plasmid	Description	Source or reference
**Strains**		
NJ-35	Wilde-type, isolated from diseased crucian carp, in China	Collected in our laboratory
SM10	*E. coli* strain, λ*pir*$^{+}$, Kan	Collected in our laboratory
ΔiolR	*iolR* deletion mutant from NJ-35	This study
CΔiolR	*ΔiolR* complemented with pMMB-*iolR*	This study
ΔU876_04005	*U876_04005* deletion mutant from NJ-35	This study
ΔiolRΔU876_04005	*U876_04005* deletion mutant from *ΔiolR*	This study
ΔiolRΔU876_13290	*U876_13290* deletion mutant from *ΔiolR*	This study
ΔiolRΔU876_13510	*U876_13510* deletion mutant from *ΔiolR*	This study
ΔiolRΔU876_17900	*U876_17900* deletion mutant from *ΔiolR*	This study
ΔiolRΔU876_19910	*U876_19910* deletion mutant from *ΔiolR*	This study
BL21(DE3)	F^{-} *ompT* $hsdS_B$ (r_B^{-} m_B^{-}) *gal dcm* (DE3)	Tiangen
**Plasmid**		
pYAK1	R6K-ori suicide vector, $SacB^{+}$, Cm	Collected in our laboratory
pET32a (+)	Amp, F1 origin, His tag	Novagen

pYAK1-*iolR*	pYAK1 carrying the flanking sequence of *iolR*, Cm	This study
pYAK1- *U876_04005*	pYAK1 carrying the flanking sequence of *U876_04005*, Cm	This study
pYAK1- *U876_13290*	pYAK1 carrying the flanking sequence of *U876_13290*, Cm	This study
pYAK1- *U876_13510*	pYAK1 carrying the flanking sequence of *U876_13510*, Cm	This study
pYAK1- *U876_17900*	pYAK1 carrying the flanking sequence of *U876_17900*, Cm	This study
pYAK1- *U876_19910*	pYAK1 carrying the flanking sequence of *U876_19910*, Cm	This study
pMMB207	Low-copy-number vector, Cm	Collected in our laboratory
pMMB-*iolR*	Plasmid pMMB207 carrying the complete ORF of *iolR*	This study

Supplementary Table 2 Primers used in this study

Primer	Sequence (5′–3′)
Cloning	
iolR-p1	CAGGTCGACTCTAGAGGATCCGCTTTGAACTGAAACAGGATT
iolR-p2	GTGATCTGCTTATGCACTCCAATCTGTTGC
iolR-p3	GGAGTGCATAAGCAGATCACAAAAATCGC
iolR-p4	GAGCTCGGTACCCGGGGATCCCGAGATACGGGACGAAAG
iolR-C-F	GAGCTCGGTACCCGGGGATCCATGAAGCTGACCCTGCAAC
iolR-C-R	CAGGTCGACTCTAGAGGATCCTCAGGTGCGGCTGGC
pet32a-*iolR*-F	GCTGATATCGGATCCGAATTCATGACAGTGGCGAAGAATCTA
pet32a-*iolR*-R	TTGTCGACGGAGCTCGAATTCTTACGAAACAATAGAGCTTTGC
P*rmaP*-F	TCCTGCTGTGGGCTGG
P*rmaP*-R	GAGGCTGGAATGGTCGGT
04005-p1	CAGGTCGACTCTAGAGGATCCATATGACGTGGTGATCTCGC
04005-p2	ATCGACCGGAGTTCTCTCCTGAATCTCTATTGG
04005-p3	AGGAGAGAACTCCGGTCGATCCCAGG
04005-p4	GAGCTCGGTACCCGGGGATCCATTCGGTAATGATGAGGTTGG
13290-p1	CAGGTCGACTCTAGAGGATCCACTGGGACAAGCCATAACC
13290-p2	AAAAATAACACATGGTTGTGTAGATAAAAAAAGA
13290-p3	CACAACCATGTGTTATTTTTCCTCAGCAGAGA
13290-p4	GAGCTCGGTACCCGGGGATCCCGTGCCGCTCAAGGAT
13510-p1	CAGGTCGACTCTAGAGGATCCTCCGCTGCCTGAACCA
13510-p2	GGAAGTTCATAAATTCTGCTTTGAGCAAAAA
13510-p3	AGCAGAATTTATGAACTTCCAACGCAGGA

13510-p4	GAGCTCGGTACCCGGGGATCCCTTCGCACCTAACGGGAC
17900-p1	CAGGTCGACTCTAGAGGATCCCAGGAATGGCTGTGGTTTG
17900-p2	GAGTAGCATAGTGAAACTGACCGTCGATCTA
17900-p3	TCAGTTTCACTATGCTACTCGCTTACTCACAA
17900-p4	GAGCTCGGTACCCGGGGATCCGCTGGCGAACACTACAAA
19910-p1	CAGGTCGACTCTAGAGGATCCCCGATGATGATGCCAAGC
19910-p2	GATATCCGCGCCATGAGGGAACCACTGC
19910-p3	TCCCTCATGGCGCGGATATCCTTGGCT
19910-p4	GAGCTCGGTACCCGGGGATCCTCGGTGGCGGTCAACTC
qRT-PCR	
iol A-F/R	CAGTCTCAGCGAGCGTATTGC / GGCTAACTCATCCCGTTTGGT
iol R-F/R	TCCTCAACCAACATTCCTCCC / CGTTTACCTTCGTTACGCACTT
iol D-F/R	GCGATTAGCGGTAGCAGTAGC / ATCAGGGGTGTAACGAGCAGG
hyp-F/R	AAACCCAACGAAGTCTGGACC / CTCCCATCGGATAACCCACTA
iol G2-F/R	CAATGAGATCGGCATGGGTAT / CAAGGTACAAAGTTGTGAAGAAGC
rbs B-F/R	ATCCCTTCAGAACGCATCCAG / CTTCCAACATTGAACGCACCA
rbs A-F/R	CACTTCTGATTTGGTCCCCAC / CTCCTGCCTTTCGTCACTGCT
rbs C-F/R	AATGATGGAGCCGATAATGGT / GCTTTATTCTCAGCGCACGTC
iol G1-F/R	GTTTTCGACGGCAATCATCTC / ATCCACTTCTACGGCACCCAC
iol C-F/R	GCAAATCCTTTCACCAGTTCG / CATCTCCCGCTTCTACAACCT
iol E-F/R	CAGTATGGGTCATCAGGTTGT / TGGAAAGAGTATGGCGAGAAG
iol B-F/R	ATCCTCACCAGTCGCAAACTC / CTAAACTCTTGTCCCGCCATC
U876_19140	GAGAACAAGACCCTGAAAGTAGG/ TAGTCGGAGAAGTTGACCACC
U876_04005	CAGCAACGGGGTGACCTACA/ TCATTCGCTTCGACCAGACG
U876_18815	CTACCCACCCACGACCTACT/ CAATGCCTTCCACTCTGATG
U876_13290	GAAAGCCTTGCTCCTTGAGT/ CTTGATCGGTCTGCTGTTGA

U876_07055	AATCTGGAACATACCAAAACTACC / ATTGACGACCTGACGAATAAA
U876_13500	GCCGTGCTCGTCGCAGTAAAT / CTCACAAGCCGATGGTGGATG
U876_17900	TGGCGAACAGTACATCAGGAG / TCAGGTGGCTATCTACGAGGC
U876_21300	GCAGCGAACGGCTGAACT / ATGGGCGGCAAGGATGAC
U876_19910	ATCCACCTTGTTGTTGAGGC / CGTCTACTACCACGGTCTGTTC
U876_13510	TTTCTCGCCTCCAGTTCACC / CCATCTCCTACCTCAAATCCC
U876_21165	CTTGACGGCATCTTCCTTGT / CGTTGCTCCTGAGTCTGTTGA
U876_09855	AAGGTCGGCTCGCCAAAGTG / CGGCAGGGAGAAGTGGAAGG
U876_09905	AAGACGGTCACCTCGCTCAG / TCCATCCATCCCTCCCTCAA
U876_09890	TCTCCAGGTTAGGGTTGTCC / GGTGGTGGTACTGAGCGAAA
U876_06550	GTGCGGGGCTTCTCGAAATA / GTCATCGGTCCCTGCTCCAT
U876_02380	AGGCACAGGTGAGGTTCTTCG / GCCAACTGGTGGGACGCTACT
U876_09570	AGATCCATATCCCTGGCAATC / GACTCTACGGCAAGCAAAACA
U876_12970	TGGTCGTAGCAGGAGTAGTAGG / ATGGCTTCGCTCTGGAGTAAC
U876_15580	GAGGATCACATCCACCCGGTTAC / GCCCCGAGCAGAGCCATTAT
U876_00930	TCTACAAATACGACGACAACTTCA / GTCATTCATCAGCAGCTCCAC

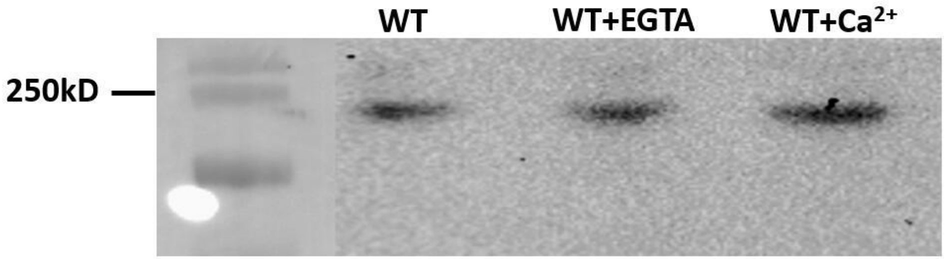

Supplementary Figure 1 The effect of Ca^{2+} on the conformation of RmpA. The conformation of RmpA was analyzed by western-blot in native gels with bacteria grown in LB medium supplemented 10mM EGTA or 10 mM $CaCl_2$. Polyclonal anti-RTX domain antibody was used to measure the migratelength of RmpA. All blots were derived from the same experiment and were processed in parallel.
